# Prognostic relevance of acquired uniparental disomy in serous ovarian cancer

**DOI:** 10.1186/s12943-015-0289-1

**Published:** 2015-02-03

**Authors:** Musaffe Tuna, Zhenlin Ju, Marcel Smid, Christopher I Amos, Gordon B Mills

**Affiliations:** Departments of Epidemiology, Unit 1340, The University of Texas MD Anderson Cancer Center, 1515 Holcombe Blvd., Houston, TX 77030-4009 USA; Departments of Bioinformatics and Computational Biology, The University of Texas MD Anderson Cancer Center, Houston, TX USA; Departments of Systems Biology, The University of Texas MD Anderson Cancer Center, Houston, TX USA; Department of Medical Oncology, Erasmus Medical Center – Daniel den Hoed Cancer Center, and Cancer Genomics Center, Rotterdam, The Netherlands

**Keywords:** Acquired uniparental disomy, Ovarian cancer, Overall survival, Recurrence-free survival

## Abstract

**Background:**

Acquired uniparental disomy (aUPD) can lead to homozygosity for tumor suppressor genes or oncogenes. Our purpose is to determine the frequency and profile aUPD regions in serous ovarian cancer (SOC) and investigated the association of aUPD with clinical features and patient outcomes.

**Methods:**

We analyzed single nucleotide polymorphism (SNP) array-based genotyping data on 532 SOC specimens from The Cancer Genome Atlas database to identify aUPD regions. Cox univariate regression and Cox multivariate proportional hazards analyses were performed for survival analysis.

**Results:**

We found that 94.7% of SOC samples harbored aUPD; the most common aUPD regions were in chromosomes 17q (76.7%), 17p (39.7%), and 13q (38.3%). In Cox univariate regression analysis, two independent regions of aUPD on chromosome 17q (A and C), and whole-chromosome aUPD were associated with shorter overall survival (OS), and five regions on chromosome 17q (A, D-G) and *BRCA1* were associated with recurrence-free survival time. In Cox multivariable proportional hazards analysis, whole-chromosome aUPD was associated with shorter OS. One region of aUPD on chromosome 22q (B) was associated with unilateral disease. A statistically significant association was found between aUPD at *TP53* loci and homozygous mutation of *TP53* (*p* < 0.0001).

**Conclusions:**

aUPD is a common event and some recurrent loci are associated with a poor outcome for patients with serous ovarian cancer.

**Electronic supplementary material:**

The online version of this article (doi:10.1186/s12943-015-0289-1) contains supplementary material, which is available to authorized users.

## Background

Ovarian cancer is the fifth most common cancer among women, with an estimated 21,880 new cases per year in the United States [1]. Ovarian cancer has the highest mortality rate of female reproductive system cancers and is the fourth leading cause of cancer-related death in women, responsible for an estimated 13,850 deaths per year in the United States [1]. The mortality rate for ovarian cancer remains high because the disease is usually diagnosed at an advanced stage when cure rates are low; the 5-year survival rate for patients with advanced disease remains approximately 30% [2].

Approximately 90% of ovarian cancers are designated epithelial ovarian cancers (EOCs) with the majority arising either from cells lining the fallopian tube or the ovarian surface; the remaining 10% of ovarian cancers are germ cell and stromal tumors [3,4]. Ovarian cancer is a heterogeneous disease at the genetic level. The most common subtype is serous carcinoma, which comprises ~60% of EOCs [5]. Serous carcinoma can be subdivided into high-grade (HG; grades 2 and 3) and low-grade (LG; grade 1) with a strong association with patient outcomes [2]. Most serous ovarian cancers are high grade accounting for the great majority of deaths from ovarian cancer. Molecular analysis has shown that LG and HG serous carcinomas harbor distinct genetic events and do not interconvert [6-9]. Therefore, LG and HG serous OCs are now thought to be different diseases with distinct molecular characteristics and behavior [10-12].

The advent of high-density single nucleotide polymorphism (SNP) arrays combined with emerging analytical tools has made it possible to identify genome-wide copy number changes and allele-specific alterations in cancer. However, little is known about the contribution of uniparental disomy (UPD) to disease outcome [13]. UPD occurs when two homologous chromosomes, or segments of chromosomes, originate from the same parent [14]. UPD can either be constitutional or acquired (aUPD) during tumor initiation and progression. Acquired uniparental disomy (aUPD), also known as copy neutral loss of heterozygosity is a relatively common event in cancer [13,15-19]. aUPD can occur in two different ways; loss of one chromosome followed by duplication of the remaining chromosome (with the homologous chromosomes arising either maternally or paternally) leads to whole-chromosome aUPD, whereas somatic recombination leads to segmental aUPD. In both cases, copy number does not change. aUPD has the potential to lead to homozygosity of existing aberrations such as mutation, deletion, methylation, histone-modification, or imprinted genes. Therefore aUPD could contribute to development and/or progression of cancer by inactivating tumor suppressor genes or doubling the copy number of oncogenic alleles.

Until now, aUPD has not been correlated with disease outcome in large scale studies of ovarian cancer. Therefore, the purpose of this study was to use a large sample set to determine the frequency of aUPD, to identify recurrent aUPD regions, and to test whether the most frequent aUPD regions are correlated with overall survival (OS) and recurrence-free survival (RFS) in serous epithelial ovarian cancer.

## Materials and methods

### Samples

We analyzed SNP array-based genotyping data from 532 tumor samples analyzed by The Cancer Genome Atlas (TCGA) project [20].Tissue sample and clinical data were retrieved from the TCGA Data Portal (http://tcga-portal.nci.nih.gov/tcga-portal). Patient demographics are summarized in Additional file 1: Table S1. The median OS time was 28.5 months (range, 0.27 to 182.7 months). RFS time was calculated from the date of diagnosis of ovarian cancer to the date of recurrence or last follow-up. OS time was calculated from the date of diagnosis of ovarian cancer to the date of death or last follow-up. Sample and clinical data were based on a November 2013 freeze from TCGA data portal. Recurrence data and vital status were available for 532 patients (Additional file 1: Table S1). Primary response to treatment, which was defined as primary therapy outcome success; progressive disease, partial response, complete response and stable disease was determined after primary surgery and subsequent adjuvant chemotherapy. Platinum sensitivity was defined as previously published [20]. The organ side was defined as bilateral, if SOC occured in both ovaries, and was defined as unilateral if SOC occured in only the right or left ovary. The mutation status of genes was retrieved from the TCGA data portal [20].

### Genomic data and analysis

Genomic data sets (CEL files) were retrieved from the TCGA data portal (http://tcga-portal.nci.nih.gov/tcga-portal). In this study, we only included genomic data from serous ovarian tumors.

### Determination of aUPD

After quality control was performed on the data using Genotyping Console software (Affymetrix), CHP files were generated. The data that passed the quality control process included 532 tumor specimens from TCGA. Copy Number Analyser for GeneChip (CNAG) version 3.4 software (http://www.genome.umin.jp) with a hidden Markov model algorithm [21] was used to identify aUPD regions. The analysis of TCGA data was done by using matching normal reference samples. In the aUPD analyses both genotype information and intensity were used. Chromosome analysis suite (ChAS) (Affymetrix) was used for validation of aUPD (Additional file 2: Figure S1). The aUPD-score was calculated by counting the total number of segmental aUPD regions (telomere and centromere) and whole chromosome aUPD in each sample. If aUPD occurs as a result of a single mitotic recombination, it is defined as telomeric, and if aUPD occurs via two or more mitotic recombination, it is defined as centromeric. The smallest overlapping regions of aUPD were situated by comparing aUPD endpoints (3′ and 5′). The May 2006 human genome browser (NCBI Build 36/hg18; http://genome.ucsc.edu) was used for identification of gene localization.

### Statistical analysis

Non-parametric Kruskal–Wallis or Wilcoxon Rank Sum tests were used to compare the frequency of total, telomeric, centromeric, segmental, and whole-chromosome aUPD between wild type and mutation, stage or grade for aUPD regions associated with outcome of SOC. An non-parametric Kruskal-Wallis test was used to evaluate the correlation of aUPD regions and mutation of *TP53*, double-strand break genes or homologous recombination genes (Additional file 3: Table S2) and frequency of aUPD. This study complied with REMARK (reporting recommendations for tumor-marker prognostic studies) criteria [22]. TCGA samples were divided in two independent sets: set A consists of batchs#9-17; 270 samples and set B batchs#18-40; 262 samples. There is no statistically difference between two groups in OS time (*p* = 0.27), RFS time (*p* = 0.20) (Additional file 4: Figure S2), age (*p* = 0.33), platinum status (*p* = 0.09), anatomic side (*p* = 0.71) and total aUPD (*p* = 0.80), except tumor stage (*p* = 4.8×10^−4^) and tumor grade (*p* = 1.4×10^−5^). The significance tests between two independent sample sets A and B were performed with the 2-sample student’s t-test for age and total aUPD, log-rank test for OS and RFS time, and with Chi-square’s test for platinum status, anatomic side, tumor stage and grade. Kaplan–Meier survival curves were used to plot RFS and OS probabilities for groups with and without aUPD. The log-rank test was used to test whether RFS and OS probability were significantly different between the groups. Univariate cox proportional hazards regression analysis (COXPH) was used to determine whether aUPD regions were associated with RFS time and/or OS time. Multivariate Cox proportional hazard model was performed to test differences in OS or RFS. Multivariable analysis was constructed including the following variables: age, stage, grade, response to primary therapy, platinum status, aUPD and mutation status of *PTEN, BRCA1*, *BRCA2, TP53*, *NF1* and *RB1* genes and UPD regions at chromosome 9q, 13q, 17p, 17q, 22q. A goodness of fit chi-squared test was performed to evaluate the association between aUPD regions at *TP53* and homozygous *TP53* mutation. A chi-squared test was also used to test the association between aUPD regions and unilateral disease and resistance to therapy. A finding was declared significant when the two-sided p-value was less than 0.05. Multiple testing used the Benjamini-Hochberg procedure [23] to control the false discovery rate (FDR) at less than 0.05. Statistical analyses were performed using R 2.14.0 (http://www.r-project.org) and STATA v10 (STATA Corp., College Station, TX).

## Results

### Frequency and distribution of genome-wide aUPD in ovarian cancer

**Figure 1:**
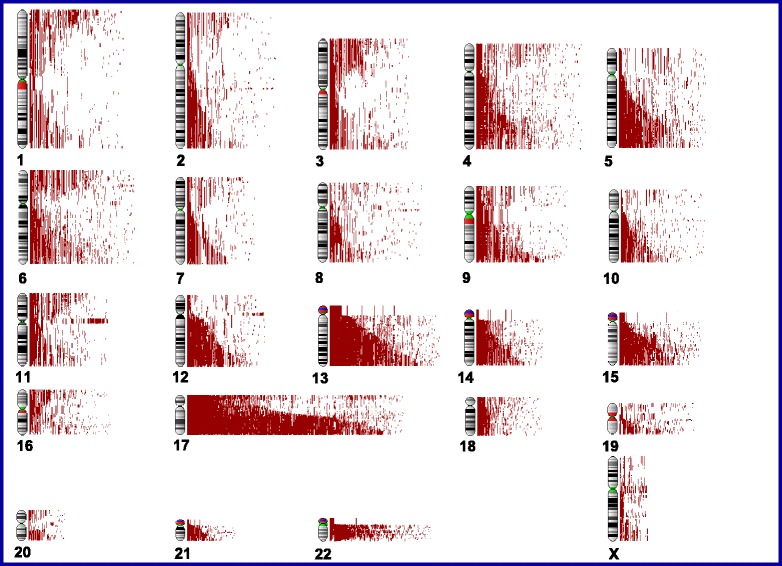
**Genome-wide profiling of aUPD regions in 532 epithelial ovarian tumor samples.** Each red line represents aUPD region for each tumor sample.

We analyzed SNP array-based genotyping data to determine the distribution and frequency of aUPD regions in epithelial ovarian tumors. Our analysis yielded a total of 5,434 aUPD regions in all chromosomes for all samples (range, 0 to 51.0 regions per sample; mean, 10.2; median, 9.0). We found that 94.7% (504/532) of ovarian cancers harbored at least one aUPD; the most common aUPD regions were in chromosomes 17q (76.7%; 408/532), 17p (39.7%; 211/532), and 13q (38.3%; 204/532), indicating that aUPD is a common event in ovarian cancer (Figure 1).

**Figure 2:**
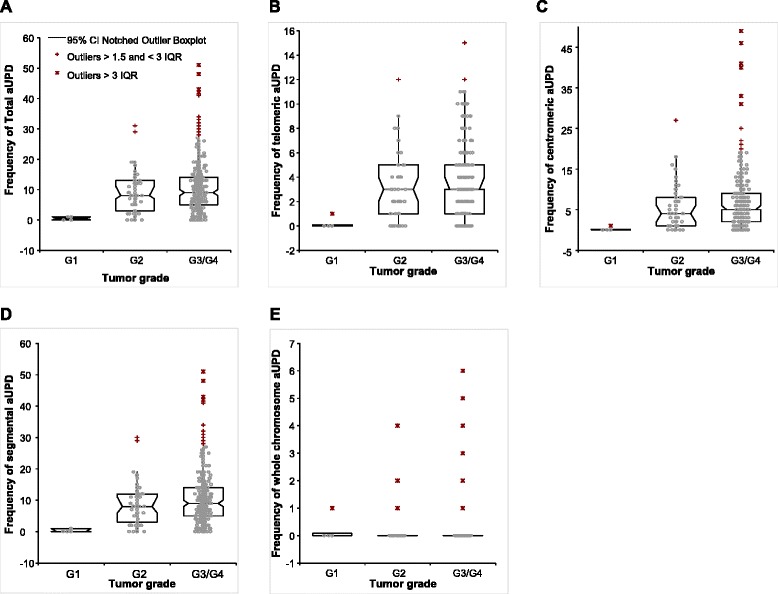
**Frequency of (A) total, (B) telomeric, (C) centromeric, (D) segmental, and (E) whole-chromosome aUPD in tumors with grade 1, 2, and 3 ovarian cancer.**

Next, we assessed whether the frequency of total aUPD varied among tumor stages and grades. In the Kruskal–Wallis test, the frequency of total aUPD in grades 2 and 3 serous ovarian cancer was significantly different from that in grade 1 (*p* = 0.0002), [(albeit with a very small number of grade 1 samples (6)] with the highest aUPD frequency in grade 3 (Figure 2A) tumors, with similar results observed when telometric (*p* = 0.0009, Figure 2B), centromeric (*p* = 0.0004, Figure 2C) and segmental (*p* = 0.0002, Figure 2D) aUPD were assessed. In contrast whole chromosome aUPD did not correlate with grade (*p* = 0.8004, Figure 2E). The aUPD score (total, telomeric, centromeric, segmental and whole chromosome) was not significantly different among stages (*p* = 0.156, *p* = 0.107, *p* = 0.144 and *p* = 0.118, *p* = 0.311, respectively) (Additional file 5: Figure S3).

aUPD can lead to homozygosity for tumor suppressor genes or oncogenes. To test for correlation between homozygous mutation and aUPD regions, we integrated the mutation status of *TP53* at chromosome 17p13.1 with aUPD as *TP53* is mutated with a high frequency in high grade serous ovarian cancer. Chi-squared analysis indicated that there was a statistically significant association (*p* < 0.0001) between homozygous mutation of *TP53* and aUPD at chromosome 17p13.1 supporting a role for aUPD in inactivation of this tumor suppressor function in high grade SOC.

### Recurrent aUPD regions and association with OS time and RFS time

First, we determined the smallest overlapping regions (SORs) in chromosomes with frequent aUPD regions: chromosomes 17q (8 independent regions, A–H), 17p (5 independent regions, A–E), 13q (2 independent regions, A and B), 9q (2 independent regions, A and B), and 22q (4 independent regions, A–D) (Additional file 6: Table S3). Then, we tested whether any of these SORs were associated with OS and/or RFS.

**Figure 3:**
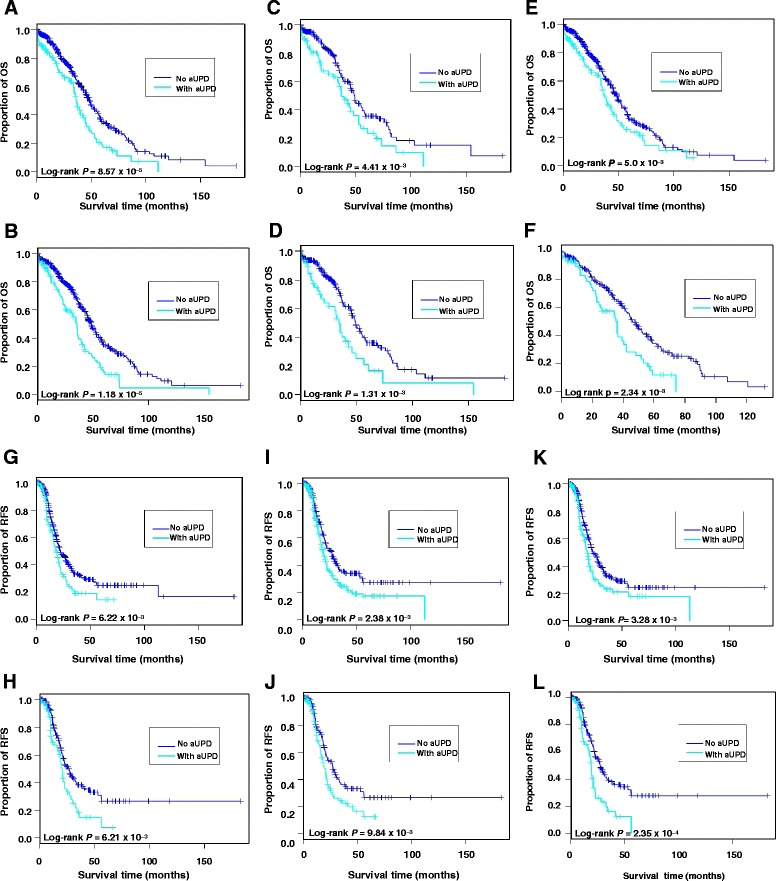
**Overall survival and recurrence-free survival analyses.** Kaplan–Meier plot of overall survival probability as a function of time for patients with aUPD at chromosome **(A)** 17q A, **(B)** whole chromosome in all tumor samples, **(C)** 17q A, **(D)** whole chromosomes in samples set B and **(E)** 17q C in all samples , and **(F)** whole-chromosome in sample set A. Kaplan-Meier plot of recurrence-free survival probability as a function of time for patients with aUPD at chromosome **(G)** 17q A, **(I)** 17q D **(K)** *BRCA1* loci in all samples, and **(H)** 17q A, **(J)** 17q D and **(L)** *BRCA1* loci in sample set B. Patients at risk at various time points are indicated.

Univariate analysis demonstrated that two regions at chromosome 17q (A) (24.2%) (*q* = 0.0009, Benjamini-Hochberg’s FDR) (Figure 3A) and (C) (24.6%) (*q* = 0.03) (Figure 3E), and samples harboring whole-chromosome aUPD (19.9%) (*q* = 0.0002) (Figure 3B) were associated with a shorter OS time (Table 1, Additional file 6: Table S3), while five regions of aUPD at 17q (A, D, E, F, G) were associated with shorter RFS time (*q* = 0.03, *q* = 0.02, *q* = 0.008, *q* = 0.02, *q* = 0.01, and *q* = 0.02, respectively) (Figure 3G-I, Table 1, and Additional file 6: Table S3, Additional file 7: Figure S4). As expected from previous studies [20], univariate analysis showed that platinum sensitivity was associated with OS and RFS (Table 1, Additional file 7: Figure S4). However, no significant correlation was found between aUPD regions and platinum status. We also determined whether aUPD at *NF1*, *RB1*, *BRCA1*, *BRCA2*, and *PTEN,* which are mutated or homozygously deleted at a low prevalence in high grade SOC [20], were associated with OS time and RFS time. Univariate analysis demonstrated that aUPD at *BRCA1* loci was associated with shorter RFS time in all samples (*q* = 0.02) (Figure 3K and Table 1), but not with OS time. The results of multivariate analysis showed that only aUPD at whole chromosomes (*p* = 0.011) and platinum sensitivity (*p* = 2.37×10^−7^) were significant prognostic factors contributing to OS time, with platinum status (*p* = 2×10^−16^) being a significant predictor of RFS (Table 2) in all samples.

**Table 1:** **Univariate analysis of clinical and genetic factors**

**Variable**	**OS**											
	**Sample set A**				**Sample set B**				**All samples**			
	**HR**	**95% CI**	***p***	***q***	**HR**	**95% CI**	***p***	***q***	**HR**	**95% CI**	***p***	***q***
Age >50 vs <50	1.36	0.87-2.12	0.18	0.52	1.62	1.05-2.52	**0.031**	0.14	1.50	1.09-2.04	**0.011**	0.058
Stage, I&II vs III&IV	0.89	0.33-2.41	0.82	0.89	5.80	2.14-15.70	**0.0005**	**0.007**	3.15	1.56-6.37	**0.001**	**0.010**
Grade, 1&2 vs 3&4	1.49	0.80-2.79	0.21	0.52	1.50	0.96-2.33	0.073	0.21	1.48	1.04-2.12	**0.032**	0.097
Platinum status,												
**Resistance vs sensitive**	5.28	3.23-8.64	**3.41×10** ^**−11**^	**1.26×10** ^**−9**^	3.29	2.07-5.22	**4.28×10** ^**−7**^	**1.58×10** ^**−5**^	4.02	2.90-5.57	**2×10** ^**−16**^	**2×10** ^**−16**^
Organ side,												
Bilateral vs unilateral	1.39	0.93-2.1	0.11	0.45	0.98	0.67-1.45	0.92	0.92	1.15	0.87-1.53	0.32	0.44
aUPD regions												
**17q A**	1.73	1.16-2.58	**0.007**	0.06	1.67	1.17-2.39	**0.005**	**0.03**	1.69	1.30-2.20	**0.0001**	**0.0009**
17q B	1.40	0.94-2.09	0.099	0.45	1.39	0.96-2.01	0.080	0.21	1.39	1.06-1.81	**0.017**	0.07
**17q C**	1.51	1.02-2.23	**0.042**	0.31	1.44	1.00-2.07	0.051	0.19	1.46	1.12-1.90	**0.005**	**0.03**
17q D	1.25	0.89-1.77	0.202	0.52	1.38	0.99-1.92	0.059	0.20	1.32	1.04-1.68	**0.021**	0.08
17q E	1.17	0.83-1.65	0.365	0.70	1.40	1.01-1.96	**0.046**	0.19	1.29	1.01-1.63	**0.039**	0.10
22q D	1.43	0.75-2.74	0.280	0.65	2.23	1.09-4.60	**0.029**	0.14	1.68	1.04-2.71	**0.035**	0.10
aUPD regions												
*PTEN*	1.66	0.98-2.80	0.059	0.37	1.51	0.83-2.73	0.175	0.34	1.56	1.06-2.32	**0.025**	0.08
*NF1*	1.44	0.96-2.16	0.074	0.39	1.40	0.94-2.07	0.094	0.22	1.43	1.08-1.89	**0.012**	0.06
**Whole chromosome**												
aUPD	1.80	1.23-2.65	**0.003**	**0.03**	1.89	1.27-2.81	**0.002**	**0.01**	1.83	1.39-2.41	**1.59×10** ^**−5**^	**0.0002**
	**RFS**											
Age >50 vs <50	1.25	0.83-1.88	0.283	0.62	1.57	1.03-2.38	**0.037**	0.11	1.39	1.04-1.87	**0.026**	0.08
Stage, I&II vs III&IV	0.48	0.18-1.31	0.151	0.54	2.58	1.39-4.79	**0.003**	**0.02**	1.85	1.10-3.11	**0.021**	0.07
Grade, 1&2 vs 3&4	2.26	1.10-4.63	0.026	0.49	1.10	0.73-1.66	0.639	0.81	1.34	0.95-1.90	0.093	0.23
Platinum status,												
**Resistance vs sensitive**	2.63	1.75-3.95	**3.15×10** ^**−6**^	**0.0001**	9.04	5.76-14.17	**2×10** ^**−16**^	**2×10** ^**−16**^	3.84	2.88-5.13	**2×10** ^**−16**^	**2×10** ^**−16**^
Organ side												
Bilateral vs unilateral	1.27	0.85-1.91	0.245	0.61	1.36	0.90-2.05	0.148	0.39	1.30	0.97-1.73	0.076	0.20
aUPD regions												
**17q A**	1.26	0.84-1.90	0.263	0.61	1.68	1.15-2.44	**0.007**	**0.03**	1.47	1.11-1.93	**0.007**	**0.03**
17q B	1.60	0.77-1.75	0.482	0.78	1.73	1.18-2.52	**0.005**	**0.02**	1.41	1.07-1.87	**0.014**	0.06
17q C	1.14	0.75-1.72	0.535	0.78	1.73	1.18-2.52	**0.005**	**0.02**	1.41	1.06-1.86	**0.016**	0.06
**17q D**	1.34	0.95-1.89	0.098	0.54	1.55	1.11-2.17	**0.010**	**0.04**	1.45	1.14-1.84	**0.003**	**0.02**
**17q E**	1.33	0.94-1.88	0.106	0.54	1.77	1.26-2.49	**0.0009**	**0.01**	1.54	1.21-1.96	**0.0005**	**0.008**
**17q F**	1.28	0.91-1.81	0.162	0.54	1.68	1.20-2.36	**0.002**	**0.02**	1.47	1.16-1.87	**0.002**	**0.02**
**17q G**	1.30	0.92-1.85	0.135	0.54	1.70	1.21-2.38	**0.002**	**0.02**	1.49	1.17-1.89	**0.001**	**0.01**
17q H	1.37	0.94-1.99	0.102	0.54	1.22	0.87-1.71	0.245	0.49	1.29	1.00-1.66	**0.044**	0.13
aUPD regions												
*PTEN*	1.10	0.61-1.54	0.761	0.88	1.32	0.67-2.61	0.419	0.57	1.17	0.75-1.83	0.495	0.72
*NF1*	1.23	0.81-1.87	0.341	0.66	1.64	1.11-2.44	**0.013**	**0.05**	1.42	1.07-1.89	**0.017**	0.06
***BRCA1***	1.13	0.76-1.67	0.548	0.78	1.97	1.36-2.84	**0.0003**	**0.006**	1.49	1.14-1.94	**0.003**	**0.02**
Whole chromosome												
aUPD	1.16	0.77-1.75	0.473	0.77	1.22	0.78-1.89	.379	0.57	1.18	0.88-1.59	0.275	0.64

*Abbreviation:* *OS* overall survival, *RFS* recurrence free survival, *aUPD* acquired uniparental disomy, *HR* hazard ratio, *q* Benjamini-Hochberg’s FDR. q < 0.05 was used to select features; bold indicates statistically significant variables.

**Table 2:** **Multivariate analysis of clinical and genetic factors**

**Variable**	**HR**	**95% CI**	***p***
**OS (all samples)**			
Platinum status,			
Resistance vs sensitive	3.27	2.09-5.12	2.37×10^−7^
aUPD at			
whole-chromosome	1.82	1.15-2.88	0.0108
**OS (sample set A)**			
Platinum status,			
Resistance vs sensitive	5.14	2.81-9.4	1.01×10^−7^
aUPD at			
whole chromosome	2.17	1.29-3.66	0.0036
**OS (sample set B)**			
Platinum status,			
Resistance vs sensitive	2.48	1.12-5.47	0.0246
**RFS (all samples)**			
Platinum status,			
Resistance vs sensitive	4.16	3.07-5.64	2×10^−16^
**RFS (sample set A)**			
Platinum status			
Resistance vs sensitive	2.93	2.00-4.30	3.24×10^−8^
**RFS (sample set B)**			
Platinum status,			
Resistance vs sensitive	58.42	19.22-171.29	1.25×10^−13^

*Abbreviation:* *OS* overall survival, *RFS* recurrence free survival, *aUPD* acquired uniparental disomy, *HR* hazard ratio, q < 0.05 was used to select features.

We split the set into two parts (Set A and Set B) to provided independent sets for analysis. Univariate analysis demonstrated that one region at chromosome 17q (A) (*q* = 0.03) (Figure 3C) and samples harboring whole-chromosome aUPD (*q* = 0.01) (Figure 3D) were associated with shorter OS in the sample set B, and only samples with whole-chromosome aUPD (*q* = 0.03) (Figure 3F) were associated with shorter OS time in set A (Table 1). In addition, aUPD in seven regions at 17q (A-G) (*q* = 0.03, *q* = 0.02, *q* = 0.02, *q* = 0.04, *q* = 0.01, *q* = 0.02, and *q* = 0.02, respectively), and aUPD at *NF1* (*q* = 0.05), and *BRCA1* loci (*q* = 0.006) (Figure 3H, J and L, Table 1, Additional file 7: Figure S4) were associated with shorter RFS in set B, but not in set A. Univariate analysis showed that platinum sensitivity was associated with shorter OS in both sets A and B (*q* = 1.26×10^−9^ and *q* = 1.58×10^−5^, respectively), and shorter RFS in both sets (*q* = 0.0001 and *q* = 2×10^−16^, respectively) (Table 1, Additional file 7: Figure S4). The results of multivariate analysis showed that only platinum sensitivity was significant predictor of OS time in both sets A (*p* = 1.01×10^−7^) and B (*p* = 0.025), while whole-chromosome aUPD was significant predictor of OS time in set A (*p* = 0.004) (Table 2), but not in set B, with platinum status being a significant predictor of RFS in set A (*p* = 3.24×10^−8^) and B (*p* = 1.25×10^−13^). After that analysis was performed, we also combined set A and B to determine if with the increased power we could identify potential predictors. However, in the merged set, we did not have an independent test set and all analysis should be considered exploratory.

### Additional associations with aUPD regions

Chi-square analysis demonstrated that aUPD at chromosome 22q (B) (*p* = 0.0007, *q* = 0.02) was associated with unilateral ovarian tumors. In addition, the frequency of aUPD was significantly higher in ovarian cancer samples with *TP53* mutations, with aUPD at *TP53*, *BRCA1* or *BRCA2* loci, with aUPD on 17q (A-H) or 22q (B) regions (Additional file 8: Table S4 and Additional file 9: Figure S5). In contrast, no association was found between the frequency of aUPD and mutation of other genes proposed to contribute to homologous recombination or double-strand break repair (Additional file 8: Table S4).

Of the 8 regions associated with outcomes or clinical characteristics, four in chromosome 17q (A–D) and one region in chromosome 22q (B) harbor open reading frames for known proteins. Reminiscent of a number of sites identified in genome-wide association studies, the remaining 3 regions in 17q (E–G) do not harbor open reading frames for proteins (Table 3). When we integrated mutation data from the TCGA database [20] for genes known to be mutated in ovarian cancer, from the 74 genes located within the SOR aUPD regions associated with outcomes, 33 genes were mutated either homozygously or heterozygously, and 6 genes were mutated homozygously in at least one patient in the TCGA ovarian cancer database [20] (Table 3) (*p* = 0.462). Genes or potentially miRNA or noncoding RNA in SOR aUPD regions may also be inactivated by additional mechanisms such as methylation or histone modification.

**Table 3:** **Genes in the small overlapping regions of aUPD in chromosomes 17q, and 22q that correlate with OS and/or RFS time or anatomic side**

**Chromosomal region**	**Chromosomal position**	**Start-end position**	**Genes**
17q A	17q12-q21.2	35.139.198-35.611.217	***GRB7*** *,* ***IKZF3*** *,* ***ZPBP2*** *,* ***GSDMB*** *, ORMDL3, LRRC3C, GSDMA,* ***PSMD3, CSF3*** *,* ***MED24, THRA*** *, NR1D1, MSL1,* ***CASC3*** *,* ***RAPGEFL1***
17q B	17q21.2	37.079.721-37.604.187	*EIF1,* ***HAP1*** *, GAST,* ***JUP*** *, LEPREL4, NT5C3L,* ***FKBP10*** *,* ***KLHL10*** *, KLHL11,* ***ACLY*** *,* ***TTC25*** *, CNP,* ***DNAJC7, NKIRAS2*** *, ZNF385C, DHX58,* ***KAT2A*** *, HSPB9, RAB5C, KCNH4, HCRT, GHDC*
17q C	17q21.2	37.244.697-37.559.796	*NT5C3L,* ***FKBP10*** *,* ***KLHL10,*** **KLHL11** *,* ***ACLY, TTC25*** *, CNP,* ***DNAJC7, NKIRAS2*** *, ZNF385C, DHX58,* ***KAT2A*** *, HSPB9, RAB5C*
17q D	17q21.33	45.311.230-45.815.839	*DLX4, DLX3, LOC284080, ITGA3, PDK2, PPP1R9B,* ***SGCA*** *, HILS1,* ***COL1A1*** *, TMEM92, XYLT2, MRPL27,* ***EME1*** *, LRRC59*
17q E	17q22	48.041.053-43.303.286	No gene
17q F	17q22	49.352.217-49.666.897	No gene
17q G	17q22	49.771.790-50.243.809	No gene
22q B	22q11.2	29.263.787-29.738.848	*SEC14L6, GAL3ST1, PES1,* ***SLC35E4*** *, TCN2, DUSP18,* ***OSBP2*** *,* ***MORC2*** *, TUG1*

Bold indicates genes that mutated. aUPD, acquired uniparental disomy; OS, overall survival.

## Discussion

In this study, we have determined the distribution and frequency of aUPD regions in ovarian cancer and investigated the association of aUPD with RFS time and OS time. aUPD regions were found in all chromosomes, with the most frequent aUPD at chromosome 17q (76.7% of SOC samples). Pederson et al. found an increase in copy-neutral LOH in association with age [24]. The frequency of centromeric, telomeric, and segmental aUPD was higher in grades 2 and 3 than that in grade 1 SOC, consistent with previous studies indicating that grade 2 and 3 serous tumors arise from different molecular pathways [6-9].

The association between aUPD at the *TP53* region and homozygous mutation of *TP53* supports the contention that aUPD can lead to inactivation of the function of important tumor suppressor genes in SOC. Tumors harboring aUPD at *TP53*, 17q A-H, or 22q B and D regions were associated with a higher frequency of total aUPD events suggesting that *TP53* as well as genes in the 17q and 22q regions may contribute or select for aUPD.

Two regions at chromosome 17q (A and C) and whole-chromosome aUPD were associated with shorter OS time, and five regions of aUPD at 17q (A, D-G) and the *BRCA1* loci were associated with shorter RFS time in all samples (Figure 3). Previous studies showed that altered expression and copy number of genes and/or miRNAs [20,25-27] were associated with outcome of disease dependent on mechanisms of inactivation or activation of genes [20]. For instance, *BRCA1/2* mutated cases have been reported to be associated with improved OS compared to *BRCA1*/*2* wild-type, whereas methylated *BRCA1* cases had similar OS time to *BRCA1/2* wild-type cases [20]. On the other hand, LOH analysis of epithelial ovarian cancer has shown that LOH at 22q13.31-q13.33 predicts prolonged progression free survival (*p* = 0.03), that was not statistically significant in adjusted (for stage, optimal cytoreduction, and germline *BRCA* mutation status) analysis (*q* = 0.2) [13]. In addition, LOH profiling of high-grade serous ovarian cancer, separated into copy loss (hemizygosity), or copy neutral loss of one allele (homozygosity), and frequency of LOH demonstrated three subclusters (HiA, HiB and Lo) based on distribution and frequency of LOH. These three clusters were found to differ in response to chemotherapy, with the highest chemotherapy-resistance rate in the Lo-subcluster, and a longer median progression-free survival in HiA-subcluster [28]. LOH may pinpoint hemizygous or homozygous regions in the tumor genome, which often exhibit loss-of-function mutations; in contrast, aUPD pinpoints only homozygous regions, which renders both loss-of-function and gain-of-function mutations homozygous. Allele based analysis is required to distinguish between LOH and aUPD events [19,29]. Our results add novel findings to previous reports on the clinical significance of chromosomal instability in ovarian cancer: not only do chromosomal instability, LOH and gene expression profiles correlate with ovarian cancer outcome [20,25-27] but aUPD has prognostic significance in this disease.

Only aUPD at chromosome 17q was associated with shorter OS and RFS time in all samples and sample set B, and whole-chromosome aUPD was associated with shorter OS in all samples and in independent analysis of sets A and B. Thus whole-chromosome aUPD appears to be a prognostic factor in serous epithelial ovarian cancer. This led us to hypothesize that the aUPD regions in chromosome 17q harbor genes homozygous for existing abnormalities such as gain-of-function or loss-of-function mutation, methylation, histone modification, or imprinting. Indeed multiple cancer associated genes as well as genes reported to be mutated in ovarian cancers are located within these regions. Of the candidate genes in the regions, the product of *GRB7* is an adapter protein that interacts with many receptor tyrosine kinases, including the epidermal growth factor receptor (EGFR), human EGFR receptor 2 (HER2), and ephrin receptors [30]. GRB7 also plays a crucial role in the integrin signaling pathways. GRB7 is overexpressed in ovarian cancer cells and promotes cell proliferation, migration, and invasion in high grade ovarian cancer [31]. *CARD10* (also known as *CARMA3*) mediates activation of NF-κB and tumor progression [32]. Methylation of *JUP*, also known as gamma-catenin, correlates with poor prognosis in renal cell carcinoma [33]. Gamma-catenin sensitizes cells to platinum compounds with reduced levels of gamma-catenin contributing to cisplatin resistance [34]. *KAT2A* (also known as GCN5 histone acetyltransferase) controls glucose metabolism [35] and regulates cell cycle-related genes and apoptosis-related genes via histone modification [36]. *ACLY* (ATP citrate lyase) is a crucial gene in the lipogenic pathway that is overexpressed in serous ovarian cancer [37]. Inhibition of ACLY suppresses the AKT signaling pathway which is important in ovarian cancer [38].

In conclusion, the results of this study provide new insights into the role of aUPD in epithelial ovarian tumorigenesis and indicate that aUPD has prognostic relevance in this disease. Further functional studies on candidate genes in aUPD regions that have prognostic relevance will be required to elucidate their potential relevance in the pathophysiology and in treatment efficacy of ovarian cancer.

## Additional files

Additional file 1: Table S1. Tumor sample demographics used in clinical outcome. Additional file 2: Figure S1. Representative figure for segmental aUPD analyzed by (A) CNAG and (B) ChAS. Upper panel represents segmental aUPD at chromosome 8 and lower panel represents segmental aUPD at chromosome 1. Additional file 3: Table S2. Homologous recombination (HR), potential HR genes and double strand break genes. Additional file 4: Figure S2. Overall survival and recurrence-free survival analyses. Kaplan–Meier plot of (A) overall survival and (B) recurrence-free survival probability as a function of time for patients in sample set A and B. Patients at risk at various time points are indicated. Additional file 5: Figure S3. Frequency of (A) total, (B) telomeric, (C) centromeric, (D) segmental, and (E) whole-chromosome aUPD in tumors with stage I, II, III, and IV ovarian cancer. Additional file 6: Table S3. aUPD regions that associate with overall survival and/or recurrence-free survival time in all samples of serous epithelial ovarian cancer Additional file 7: Figure S4. Overall survival and recurrence-free survival analyses. Kaplan–Meier plot of recurrence free survival probability as a function of time for patients with aUPD at chromosome (A) 17q B, (B) 17q C, (C) 17q E, (D) 17q F, (E) 17q G, and (F) *NF1* loci in set B. Kaplan–Meier plot of recurrence free survival probability as a function of time for patients with aUPD at chromosome (G) 17q E, (H) 17q F, and (I) 17q G in all samples. Kaplan–Meier plot of recurrence free survival probability as a function of time for patients with platinum status in (J) set A, (K) set B, and (L) all samples. Kaplan–Meier plot of overall survival probability as a function of time for patients with platinum status in (M) set A, (N) set B, and (O) all samples. Kaplan–Meier plot of overall survival probability as a function of time for patients with response to therapy in (P) set A, (R) set B, and (S) all samples. Patients at risk at various time points are indicated. PlatinumS; platinum sensitive, PlatinumR; platinum resistance, Resistance; resistance to therapy, Response; response to therapy. Additional file 8: Table S4. Association between frequency of aUPD and mutation status of *TP53*, *BRCA1*, *BRCA2*, homolog recombination (HR) and double-strand break (DSB) genes and aUPD regions at 17q (A-H), 22q (A-D), *TP53*, *BRCA1* and *BRCA2*. Additional file 9: Figure S5. Association between frequency of aUPD and somatic mutation at *TP53*, at *BRCA1* and *BRCA2*, and aUPD at *TP53, BRCA1* and *BRCA2*. (A) Somatic mutation at *TP53*, (B) at *BRCA1* and (C) at *BRCA2*, and aUPD (D) at *TP53*, (E) at *BRCA1* and (F) at *BRCA2* loci.

## Abbreviations

aUPD: Acquired uniparental disomy
SOC: Serous ovarian cancer
SNP: Single nucleotide polymorphism
OS: Overall survival
RFS: Recurrence-free survival
HG: High-grade
LG: Low-grade
TCGA: The Cancer Genome Atlas
CNAG: Copy Number Analyser for GeneChip
REMARK: Reporting recommendations for tumor-marker prognostic studies
COXPH: Cox proportional hazards regression analysis
SORs: Smallest overlapping regions
LOH: Loss of heterozygosity

## References

[1] Jemal A, Siegel R, Xu J, Ward E (2010). Cancer statistics, 2010. CA Cancer J Clin.

[2] Kobel M, Kalloger SE, Huntsman DG, Santos JL, Swenerton KD, Seidman JD (2010). Differences in tumor type in low-stage versus high-stage ovarian carcinomas. Int J Gynecol Pathol.

[3] Feeley KM, Wells M (2001). Precursor lesions of ovarian epithelial malignancy. Histopathology.

[4] Bast RC, Hennessy B, Mills GB (2009). The biology of ovarian cancer: new opportunities for translation. Nat Rev Cancer.

[5] Lalwani N, Prasad SR, Vikram R, Shanbhogue AK, Huettner PC, Fasih N (2011). Histologic, molecular, and cytogenetic features of ovarian cancers: implications for diagnosis and treatment. Radiographics.

[6] Cho KR, Shih Ie M (2009). Ovarian cancer. Annu Rev Pathol.

[7] Singer G, Kurman RJ, Chang HW, Cho SK, Shih Ie M (2002). Diverse tumorigenic pathways in ovarian serous carcinoma. Am J Pathol.

[8] Salani R, Kurman RJ, Giuntoli R, Gardner G, Bristow R, Wang TL (2008). Assessment of TP53 mutation using purified tissue samples of ovarian serous carcinomas reveals a higher mutation rate than previously reported and does not correlate with drug resistance. Int J Gynecol Cancer.

[9] Ahmed AA, Etemadmoghadam D, Temple J, Lynch AG, Riad M, Sharma R (2010). Driver mutations in TP53 are ubiquitous in high grade serous carcinoma of the ovary. J Pathol.

[10] Malpica A, Deavers MT, Lu K, Bodurka DC, Atkinson EN, Gershenson DM (2004). Grading ovarian serous carcinoma using a two-tier system. Am J Surg Pathol.

[11] Kurman RJ, Shih Ie M (2011). Molecular pathogenesis and extraovarian origin of epithelial ovarian cancer–shifting the paradigm. Hum Pathol.

[12] Bast RC, Mills GB (2012). Dissecting “PI3Kness’: The Complexity of Personalized Therapy for Ovarian Cancer. Canc Discov.

[13] Walsh CS, Ogawa S, Scoles DR, Miller CW, Kawamata N, Narod SA (2008). Genome-wide loss of heterozygosity and uniparental disomy in BRCA1/2-associated ovarian carcinomas. Clin Cancer Res.

[14] Engel E (1980). A new genetic concept: uniparental disomy and its potential effect, isodisomy. Am J Med Genet.

[15] Tuna M, Smid M, Martens JW, Foekens JA. Prognostic value of acquired uniparental disomy (aUPD) in primary breast cancer. Breast Cancer Res Treat 2012; 132:189-96. 10.1007/s10549-011-1579-y 21604015

[16] Mohamedali AM, Smith AE, Gaken J, Lea NC, Mian SA, Westwood NB (2009). Novel TET2 mutations associated with UPD4q24 in myelodysplastic syndrome. J Clin Oncol.

[17] Raghavan M, Lillington DM, Skoulakis S, Debernardi S, Chaplin T, Foot NJ (2005). Genome-wide single nucleotide polymorphism analysis reveals frequent partial uniparental disomy due to somatic recombination in acute myeloid leukemias. Cancer Res.

[18] Sanada M, Suzuki T, Shih LY, Otsu M, Kato M, Yamazaki S (2009). Gain-of-function of mutated C-CBL tumour suppressor in myeloid neoplasms. Nature.

[19] Ha G, Roth A, Lai D, Bashashati A, Ding J, Goya R (2012). Integrative analysis of genome-wide loss of heterozygosity and monoallelic expression at nucleotide resolution reveals disrupted pathways in triple-negative breast cancer. Genome Res.

[20] The Cancer Genome Atlas (2011). Integrated genomic analyses of ovarian carcinoma. Nature.

[21] Yamamoto G, Nannya Y, Kato M, Sanada M, Levine RL, Kawamata N (2007). Highly sensitive method for genomewide detection of allelic composition in nonpaired, primary tumor specimens by use of affymetrix single-nucleotide-polymorphism genotyping microarrays. Am J Hum Genet.

[22] McShane LM, Altman DG, Sauerbrei W, Taube SE, Gion M, Clark GM (2006). REporting recommendations for tumor MARKer prognostic studies (REMARK). Breast Cancer Res Treat.

[23] Benjamini Y, Hochberg Y (1995). Controlling the false discovery rate: a practical and powerful approach to multiple testing. J R Stat Soc B.

[24] Pedersen BS, Konstantinopoulos PA, Spillman MA, De S (2013). Copy neutral loss of heterozygosity is more frequent in older ovarian cancer patients. Genes Chromosomes Cancer.

[25] Nakayama N, Nakayama K, Shamima Y, Ishikawa M, Katagiri A, Iida K (2010). Gene amplification CCNE1 is related to poor survival and potential therapeutic target in ovarian cancer. Cancer.

[26] Birrer MJ, Johnson ME, Hao K, Wong KK, Park DC, Bell A (2007). Whole genome oligonucleotide-based array comparative genomic hybridization analysis identified fibroblast growth factor 1 as a prognostic marker for advanced-stage serous ovarian adenocarcinomas. J Clin Oncol.

[27] Walsh CS, Ogawa S, Karahashi H, Scoles DR, Pavelka JC, Tran H (2008). ERCC5 is a novel biomarker of ovarian cancer prognosis. J Clin Oncol.

[28] Wang ZC, Birkbak NJ, Culhane AC, Drapkin R, Fatima A, Tian R (2012). Profiles of genomic instability in high-grade serous ovarian cancer predict treatment outcome. Clin Cancer Res.

[29] Brenton JD, Caldas C (2003). Predictive cancer genomics–what do we need?. Lancet.

[30] Daly RJ (1998). The Grb7 family of signalling proteins. Cell Signal.

[31] Wang Y, Chan DW, Liu VW, Chiu P, Ngan HY (2010). Differential functions of growth factor receptor-bound protein 7 (GRB7) and its variant GRB7v in ovarian carcinogenesis. Clin Cancer Res.

[32] Jiang T, Grabiner B, Zhu Y, Jiang C, Li H, You Y (2011). CARMA3 is crucial for EGFR-Induced activation of NF-kappaB and tumor progression. Cancer Res.

[33] Breault JE, Shiina H, Igawa M, Ribeiro-Filho LA, Deguchi M, Enokida H (2005). Methylation of the gamma-catenin gene is associated with poor prognosis of renal cell carcinoma. Clin Cancer Res.

[34] Liang XJ, Shen DW, Gottesman MM (2004). Down-regulation and altered localization of gamma-catenin in cisplatin-resistant adenocarcinoma cells. Mol Pharmacol.

[35] Lerin C, Rodgers JT, Kalume DE, Kim SH, Pandey A, Puigserver P (2006). GCN5 acetyltransferase complex controls glucose metabolism through transcriptional repression of PGC-1alpha. Cell Metab.

[36] Kikuchi H, Takami Y, Nakayama T (2005). GCN5: a supervisor in all-inclusive control of vertebrate cell cycle progression through transcription regulation of various cell cycle-related genes. Gene.

[37] Wojnarowicz PM, Breznan A, Arcand SL, Filali-Mouhim A, Provencher DM, Mes-Masson AM (2008). Construction of a chromosome 17 transcriptome in serous ovarian cancer identifies differentially expressed genes. Int J Gynecol Cancer.

[38] Furuta E, Okuda H, Kobayashi A, Watabe K (1805). Metabolic genes in cancer: their roles in tumor progression and clinical implications. Biochim Biophys Acta.

