# Supplementary material for: Prognostic relevance of acquired uniparental disomy in serous ovarian cancer
Source: Mol Cancer. 2015 Feb 3;14(1):29. doi: 10.1186/s12943-015-0289-1 (PMC4320828; doi:10.1186/s12943-015-0289-1)
Supplement: Additional file 1: Table S1. — Tumor sample demographics used in clinical outcome. [file 12943_2015_289_MOESM1_ESM.pdf]

Table S1. Tumor sample demographics used in clinical outcome

Characteristic	TCGA Sample Set A (Batch#9-17) 270	TCGA Sample Set B (Batch#18-40) 262	TCGA All Samples (Batch#9-40) 532
Age (years)			
Mean	60.2	59.2	59.7
SD	11.1	12.0	11.6
Range	34-87	26-89	26–89
Median	59	58	59
Tumor stage			
I	1 (0.4%)	14 (5.3%)	15 (2.8%)
II	8 (3.0%)	18 (6.9%)	26 (4.9%)
III	213 (78.8%)	194 (74.1%)	407 (76.5%)
IV	48 (17.8%)	33 (12.6%)	81 (15.2%)
Unknown	0 (0%)	3 (1.1%)	3 (0.6%)
Tumor grade			
1	0 (0%)	6 (2.3%)	6 (1.1%)
2	17 (6.3%)	44 (16.8%)	61 (11.5%)
3	247 (91.5%)	206 (78.6%)	453 (85.1%)
4	1 (0.4%)	0 (0%)	1 (0.2%)
Unknown	5 (1.8%)	6 (2.3%)	11 (2.1%)
Number of patients	270	262	532
Recurrent disease			
Yes	146 (54.1%)	124 (47.3%)	270 (50.8%)
No	124 (45.9%)	134 (51.2%)	258 (48.5%)
Unknown	0 (0%)	4 (1.5%)	4 (0.7%)
Mean recurrence time (days)	609.1	618.8	613.9
Median recurrence time (days)	420.5	434	426.5
Number of patients	270	262	532
Vital status			
Alive	118 (43.7%)	140 (53.4%)	258 (48.5%)
Dead	152 (56.3%)	122 (46.6%)	274 (51.5%)
Unknown	0 (0%)	0 (0%)	0 (0%)
Mean overall survival time (d)	995.2	949.9	972.8
Median overall survival time (d)	884.5	780	855
Platinum status	166	109	275
Sensitive	106 (63.9%)	81 (74.3%)	187 (68%)
Resistant	60 (36.1%)	28 (25.7%)	88 (32%)
Anatomic side			
Unilateral (left/right)	66 (24.4%)	71 (27.1%)	137 (25.7%)
Bilateral (both left and right)	186 (68.9%)	182 (69.5%)	368 (69.2%)
Unknown	18 (6.7%)	9 (3.4%)	27 (5.1%)
aUPD			
Total aUPD (median)	9	9	9
