# Supplementary figures and images for: Prognostic relevance of acquired uniparental disomy in serous ovarian cancer
Source: Mol Cancer. 2015 Feb 3;14(1):29. doi: 10.1186/s12943-015-0289-1 (PMC4320828; doi:10.1186/s12943-015-0289-1)

Figure S1

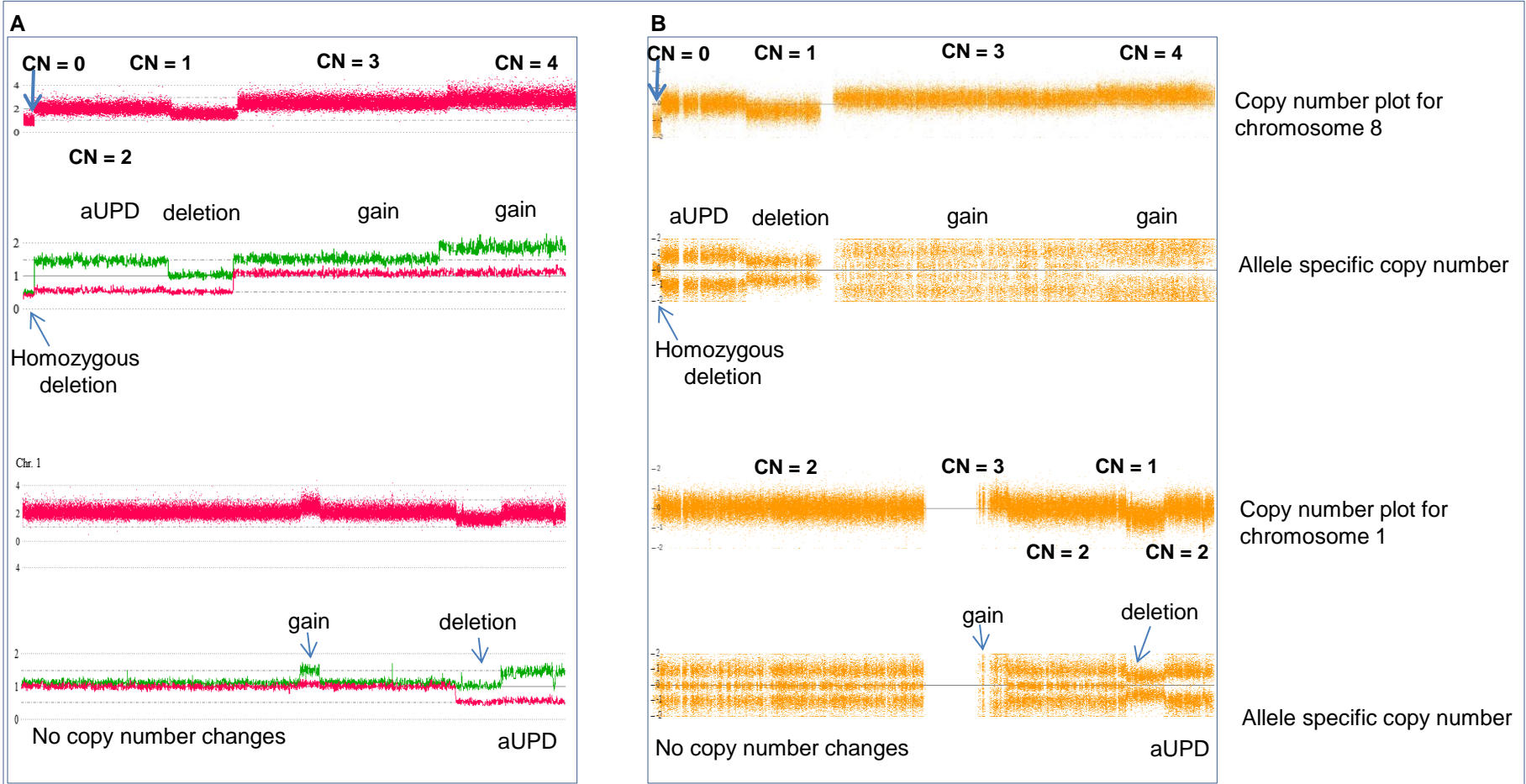

Supplement: Additional file 2: Figure S1. — Representative figure for segmental aUPD analyzed by (A) CNAG and (B) ChAS. Upper panel represents segmental aUPD at chromosome 8 and lower panel represents segmental aUPD at chromosome 1. [file 12943_2015_289_MOESM2_ESM.pdf]

Figure S2

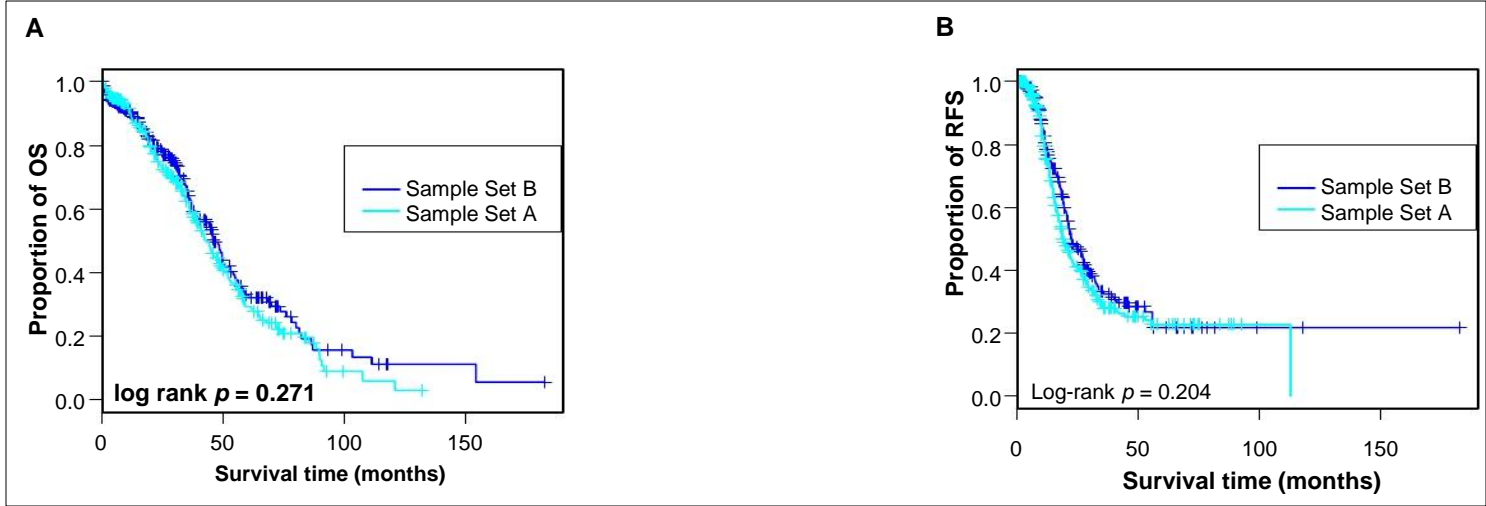

Supplement: Additional file 4: Figure S2. — Overall survival and recurrence-free survival analyses. Kaplan–Meier plot of (A) overall survival and (B) recurrence-free survival probability as a function of time for patients in sample set A and B. Patients at risk at various time points are indicated. [file 12943_2015_289_MOESM4_ESM.pdf]

**Figure S3.**

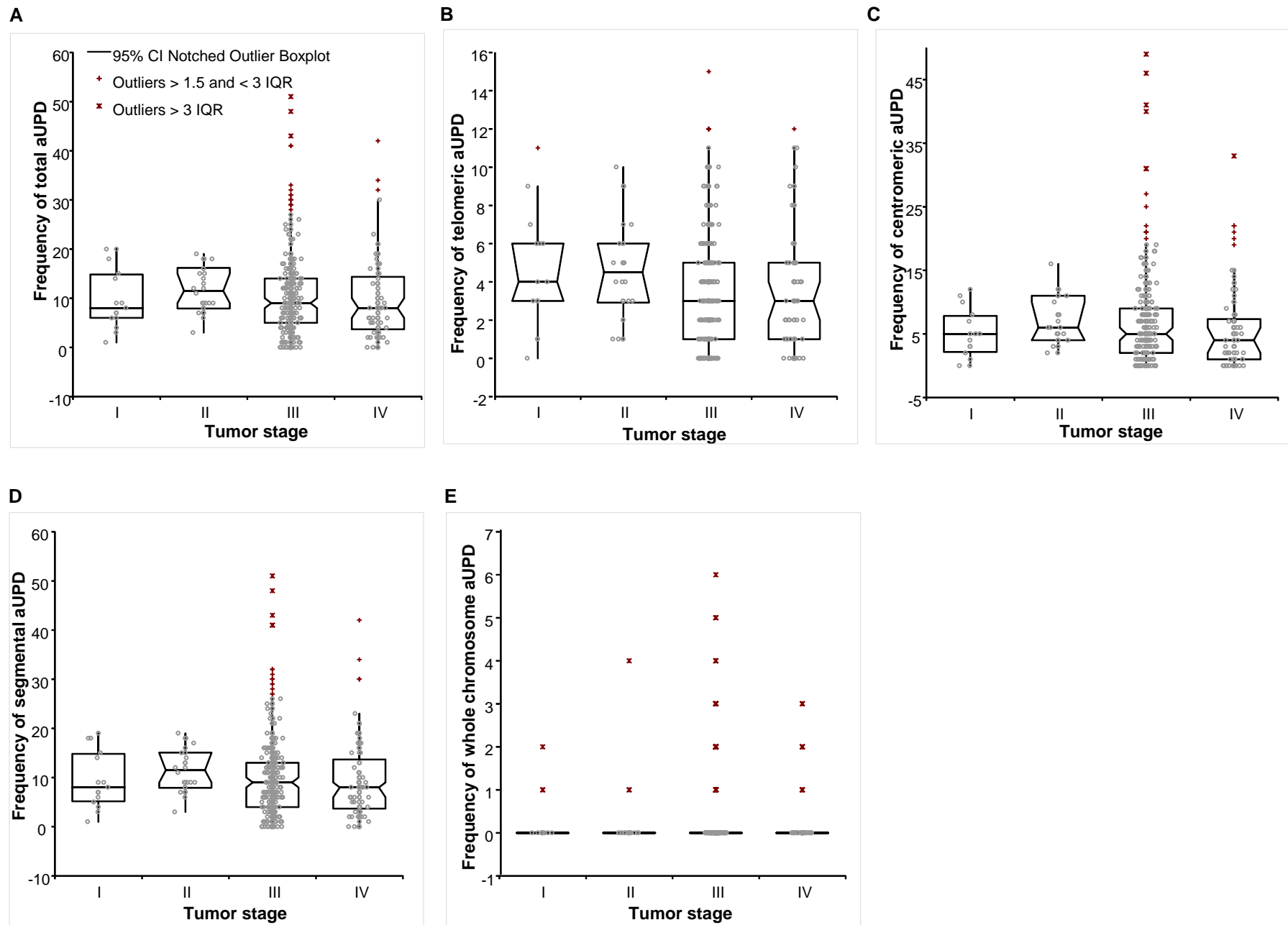

Supplement: Additional file 5: Figure S3. — Frequency of (A) total, (B) telomeric, (C) centromeric, (D) segmental, and (E) whole-chromosome aUPD in tumors with stage I, II, III, and IV ovarian cancer. [file 12943_2015_289_MOESM5_ESM.pdf]

Figure S4.

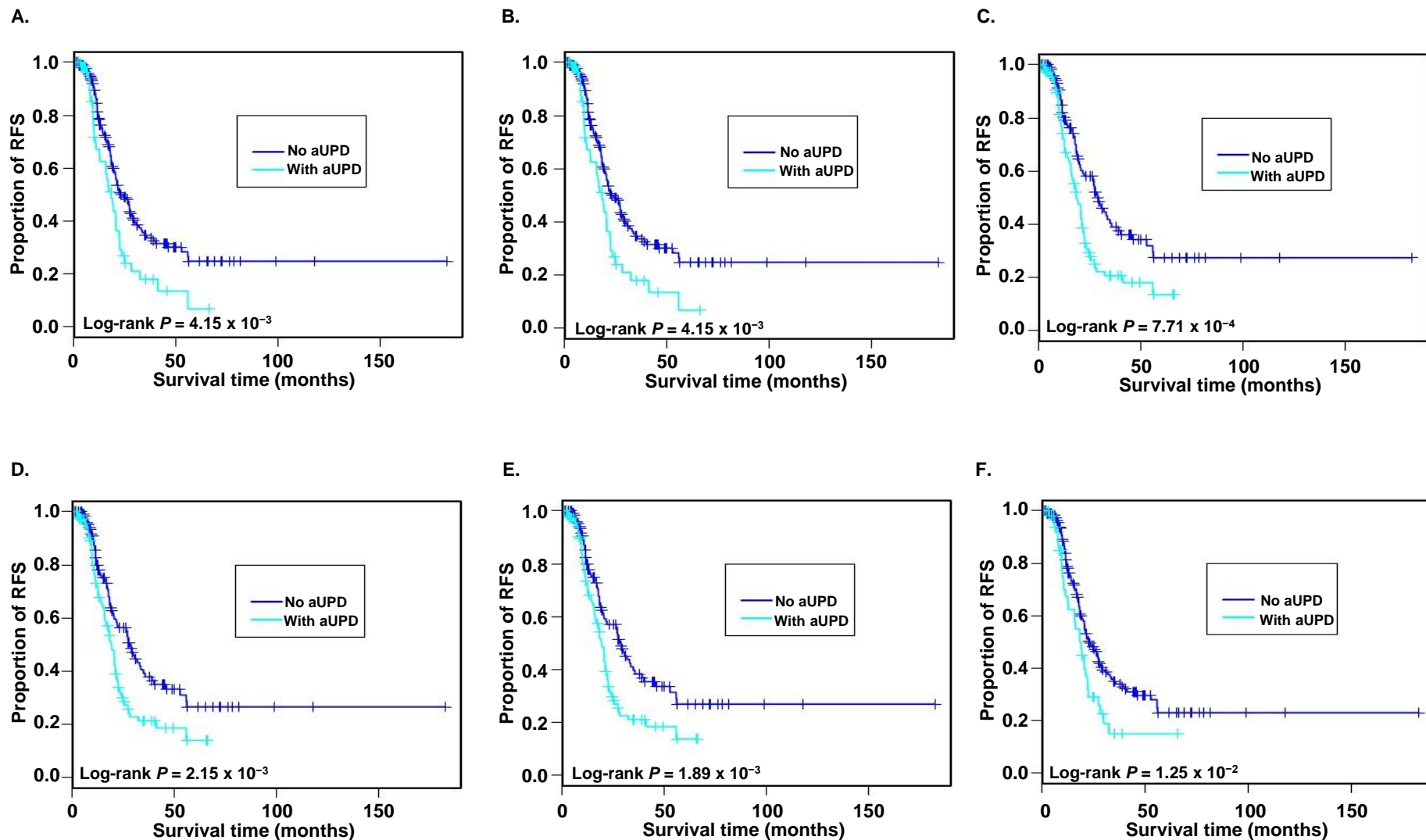

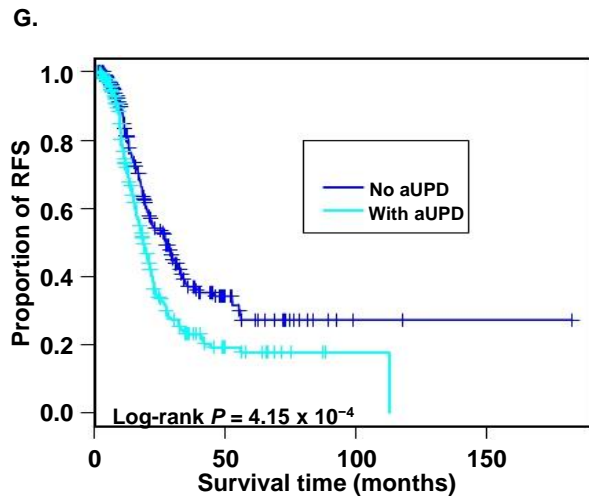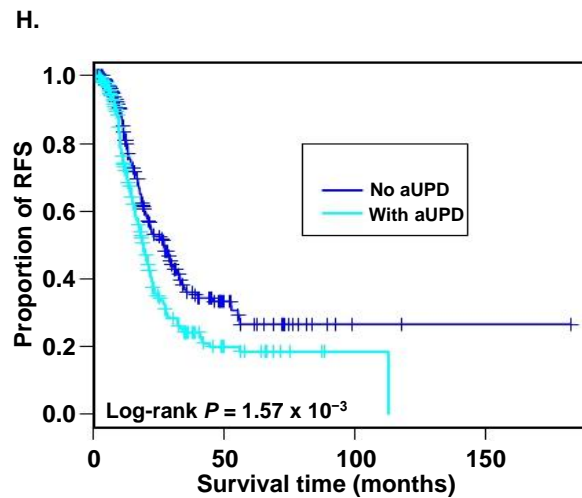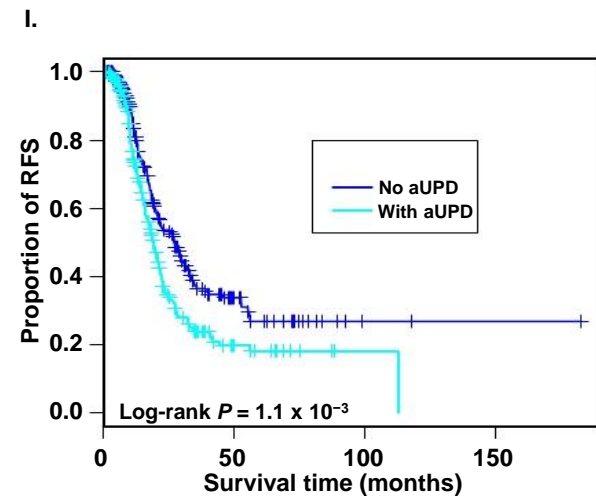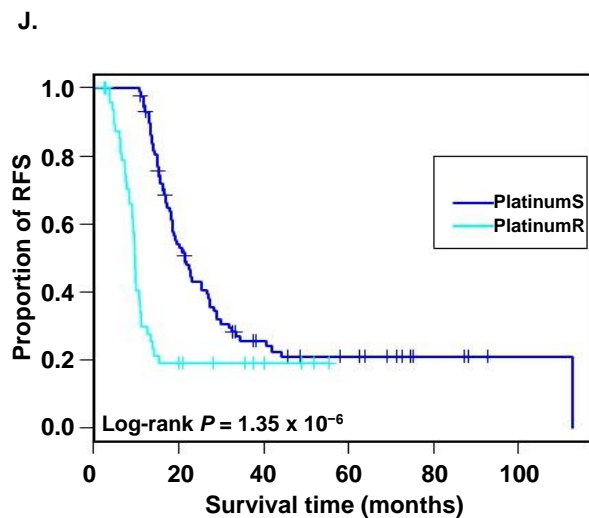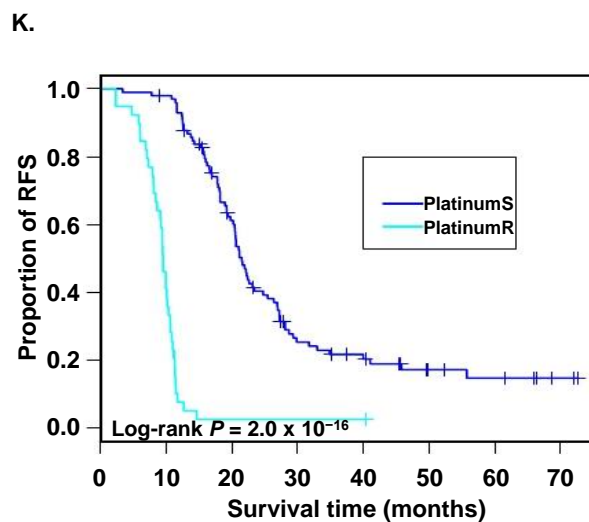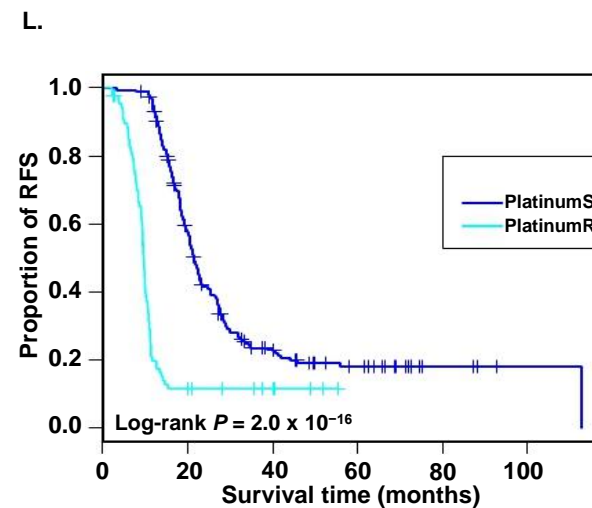

M.

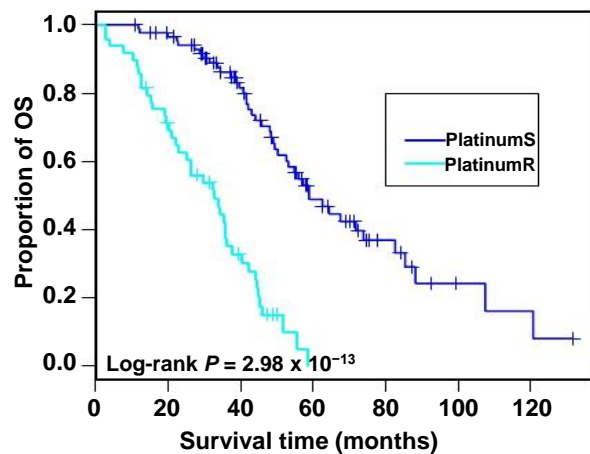

N.

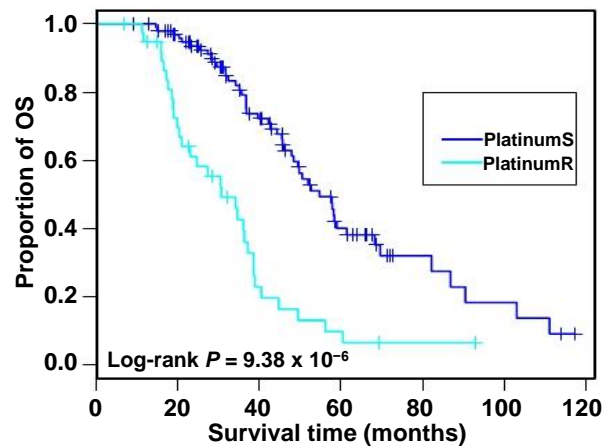

O.

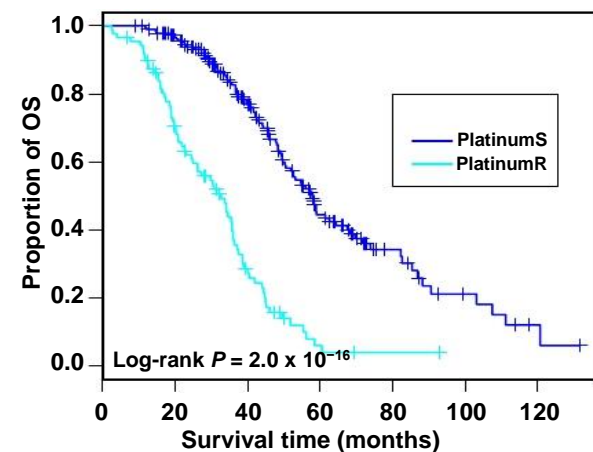

P.

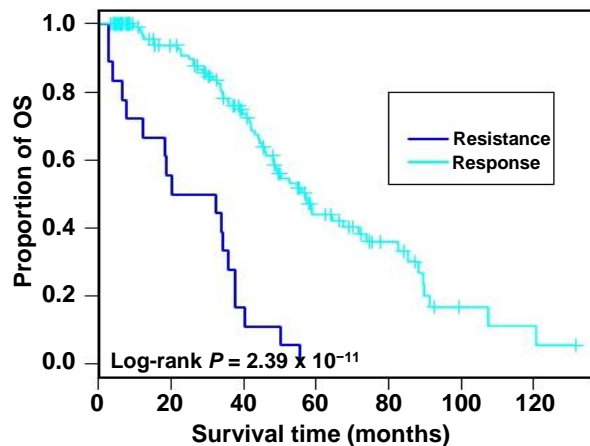

R.

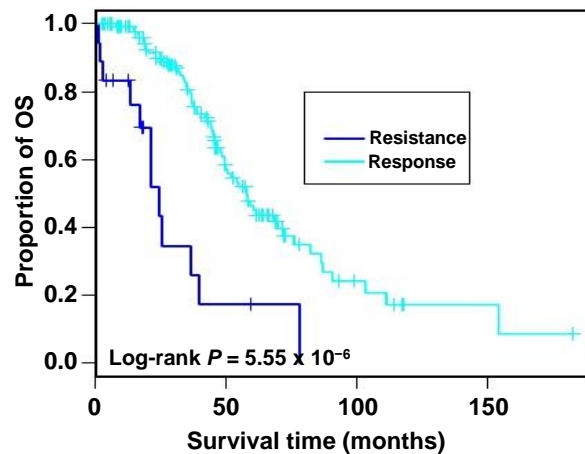

S.

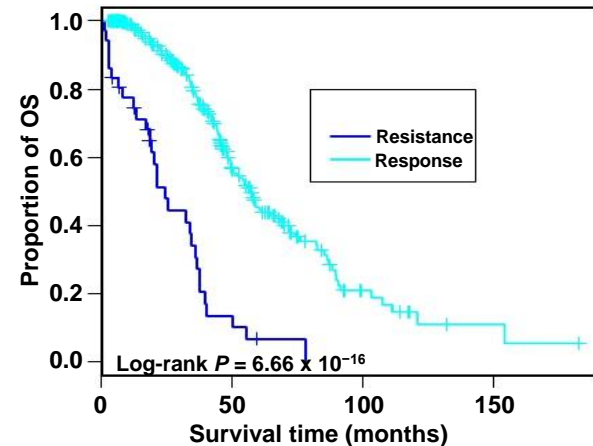

Supplement: Additional file 7: Figure S4. — Overall survival and recurrence-free survival analyses. Kaplan–Meier plot of recurrence free survival probability as a function of time for patients with aUPD at chromosome (A) 17q B, (B) 17q C, (C) 17q E, (D) 17q F, (E) 17q G, and (F) NF1 loci in set B. Kaplan–Meier plot of recurrence free survival probability as a function of time for patients with aUPD at chromosome (G) 17q E, (H) 17q F, and (I) 17q G in all samples. Kaplan–Meier plot of recurrence free survival probability as a function of time for patients with platinum status in (J) set A, (K) set B, and (L) all samples. Kaplan–Meier plot of overall survival probability as a function of time for patients with platinum status in (M) set A, (N) set B, and (O) all samples. Kaplan–Meier plot of overall survival probability as a function of time for patients with response to therapy in (P) set A, (R) set B, and (S) all samples. Patients at risk at various time points are indicated. PlatinumS; platinum sensitive, PlatinumR; platinum resistance, Resistance; resistance to therapy, Response; response to therapy. [file 12943_2015_289_MOESM7_ESM.pdf]

Figure S5.

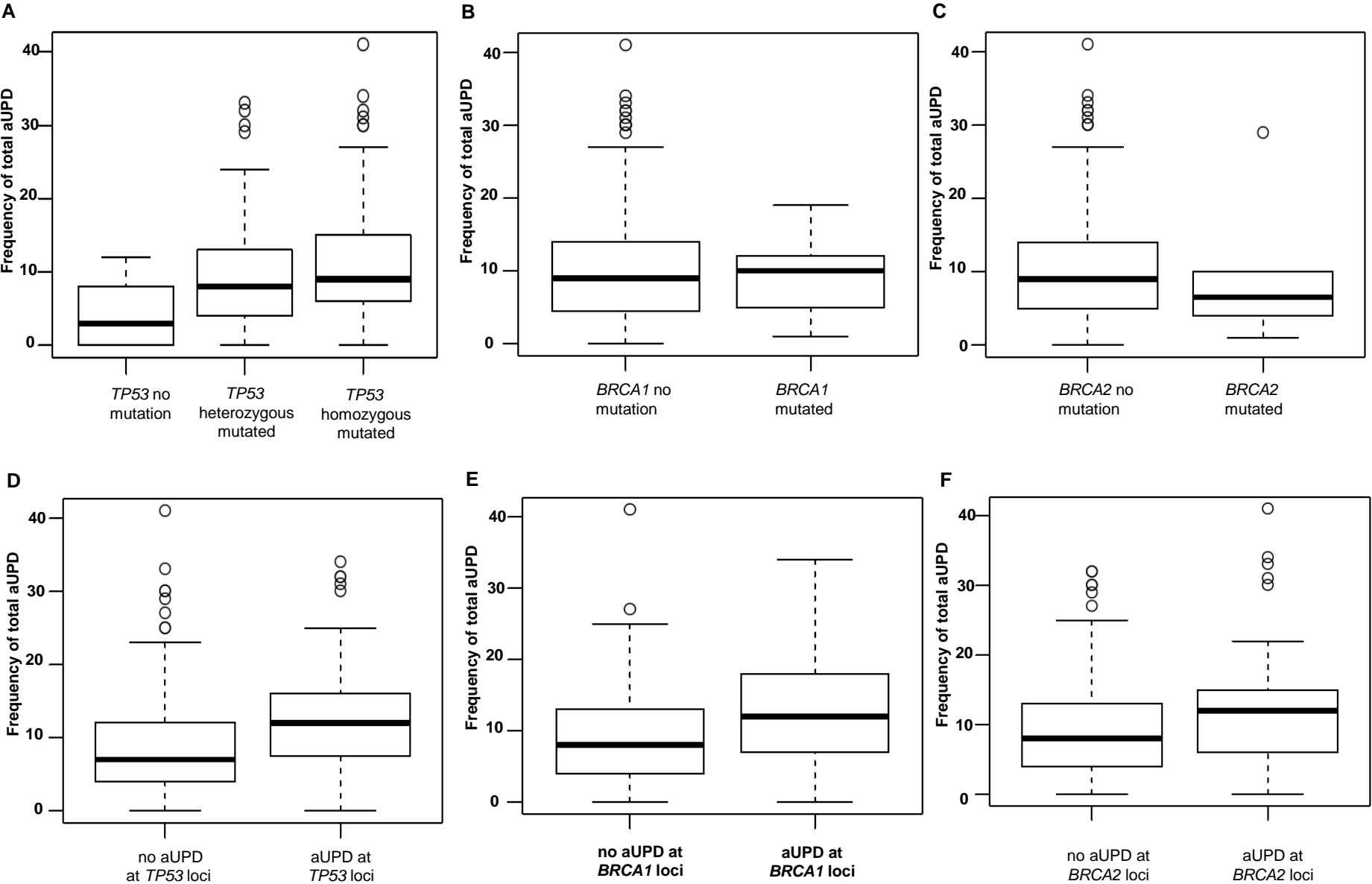

Supplement: Additional file 9: Figure S5. — Association between frequency of aUPD and somatic mutation at TP53, at BRCA1 and BRCA2, and aUPD at TP53, BRCA1 and BRCA2. (A) Somatic mutation at TP53, (B) at BRCA1 and (C) at BRCA2, and aUPD (D) at TP53, (E) at BRCA1 and (F) at BRCA2 loci. [file 12943_2015_289_MOESM9_ESM.pdf]
