# Supplementary material for: Prognostic relevance of acquired uniparental disomy in serous ovarian cancer
Source: Mol Cancer. 2015 Feb 3;14(1):29. doi: 10.1186/s12943-015-0289-1 (PMC4320828; doi:10.1186/s12943-015-0289-1)
Supplement: Additional file 3: Table S2. — Homologous recombination (HR), potential HR genes and double strand break genes. [file 12943_2015_289_MOESM3_ESM.pdf]

Table S2. Homologous recombination (HR), potential HR genes and double strand break genes.

ATM
ATR
BBC3
BCL2
BCL2L1
BLM
BRCA1
BRCA2
BRIP1
BTBD12
BUB1
BUB1B
BUB1L
BUB3
C19orf40
CD95
CDC20
CDC25C
CDC4
CENPE
CHK1
CHK2
DMC1
DNA-PK
DR5
E2F1
EME1
EME2
FANCA
FANCB
FANCC
FANCD2
FANCE
FANCF
FANCG
FANCI
FANCL
FANCM
GEN1
GIYD1
H2AFX
HAUSP
HUS1
HUS1B
KUB3 (KU70)
LIG1
LIG4
MAD1
MAD1L1
MAD2
MAD2L1
MAD2L2
MDC1
MDM2
MRE11A
MUS81
NBN
NBS1
OBFC2A
OBFC2B
P53AIP1
PALB2
PCNA
PLK1
PLK2
PLK3
PMAIP1
POLD1
POLD2
POLD3
POLD4
PTEN
RAD1
RAD17
RAD50
RAD51
RAD51C
RAD51L1
RAD51L3
RAD52
RAD54B
RAD54L
RAD9A
RB1
RBBP8
RPA1
RPA2
RPA3
RTEL1
SCOTIN
SECURIN
SHFM1
STK6
TEX15
TP53
TP53BP1
TREX1
UBE2N
XRCC2
XRCC3
XRCC4
