# Supplementary material for: Prognostic relevance of acquired uniparental disomy in serous ovarian cancer
Source: Mol Cancer. 2015 Feb 3;14(1):29. doi: 10.1186/s12943-015-0289-1 (PMC4320828; doi:10.1186/s12943-015-0289-1)
Supplement: Additional file 6: Table S3. — aUPD regions that associate with overall survival and/or recurrence-free survival time in all samples of serous epithelial ovarian cancer [file 12943_2015_289_MOESM6_ESM.pdf]

Table S3. aUPD regions that associate with overall survival and/or recurrence-free survival time in all samples of serous epithelial ovarian cancer

	OS				RFS			
**aUPD Region**	HR	95% CI	*p*	*q*	HR	95% CI	*p*	*q*
9q33.2-A	1.37	0.87-2.14	0.17	0.32	1.01	0.62-1.65	0.97	0.98
9q34.13-B	1.02	0.66-1.59	0.92	0.92	0.89	0.56-1.40	0.61	0.78
13q33.2-A	0.85	0.65-1.12	0.24	0.36	0.89	0.67-1.18	0.43	0.66
13q34-B	0.88	0.67-1.15	0.33	0.44	0.91	0.69-1.20	0.51	0.72
17p13.3-A	0.83	0.64-1.09	0.18	0.32	0.99	0.77-1.29	0.96	0.98
17p13.3-B	0.85	0.66-1.11	0.24	0.36	0.92	0.71-1.20	0.55	0.73
17p13.3-p13.2-C	0.90	0.69-1.17	0.44	0.54	1.01	0.78-1.31	0.95	0.98
17p13.1-D	0.93	0.71-1.22	0.60	0.67	1.13	0.87-1.47	0.37	0.64
17p13-E	1.01	0.76-1.35	0.92	0.92	1.05	0.79-1.39	0.75	0.90
17q12-q21.2-A	1.69	1.30-2.20	**0.0001**	**0.0009**	1.47	1.11-1.93	**0.007**	**0.03**
17q21.2-B	1.39	1.06-1.81	**0.02**	0.07	1.42	1.07-1.87	**0.01**	0.06
17q21.2-C	1.46	1.12-1.90	**0.005**	**0.03**	1.41	1.06-1.86	**0.02**	0.06
17q21.33-D	1.32	1.04-1.68	**0.02**	0.07	1.45	1.14-1.84	**0.003**	**0.02**
17q22-E	1.29	1.01-1.63	**0.04**	0.10	1.54	1.21-1.96	**0.0005**	**0.008**
17q22-F	1.24	0.98-1.57	0.08	0.18	1.47	1.16-1.87	**0.002**	**0.02**
17q22-G	1.26	0.99-1.59	0.06	0.15	1.49	1.17-1.89	**0.001**	**0.01**
17q25.3-H	1.11	0.87-1.43	0.40	0.50	1.29	1.01-1.66	**0.04**	0.13
22q11.22-A	1.12	0.75-1.66	0.59	0.67	0.83	0.54-1.28	0.40	0.64
22q11.2-B	1.08	0.75-1.57	0.67	0.73	0.82	0.56-1.22	0.33	0.64
22q13.1-C	1.40	0.94-2.09	0.10	0.21	0.92	0.59-1.42	0.70	0.86
22q13.31-D	1.68	1.04-2.71	**0.03**	0.10	0.75	0.42-1.35	0.34	0.64
PTEN	1.56	1.06-2.32	**0.03**	0.08	1.17	0.75-1.83	0.50	0.72
TP53	0.85	0.65-1.11	0.23	0.36	1.14	0.89-1.48	0.30	0.64
BRCA2	1.28	0.94-1.74	0.12	0.23	0.98	0.70-1.37	0.91	0.98
RB1	1.17	0.86-1.58	0.32	0.44	1.00	0.73-1.36	0.98	0.98
NF1	1.43	1.08-1.89	**0.01**	0.06	1.42	1.07-1.89	**0.02**	0.06
BRCA1	1.18	0.91-1.54	0.21	0.35	1.49	1.14-1.94	**0.003**	**0.02**
Whole chromosome	1.83	1.39-2.41	$\mathbf{1.59 \times 10^{-5}}$	**0.0002**	1.18	0.88-1.59	0.28	0.64

HR, hazard ratio; *q*, Benjamini-Hochberg's FDR. $q < 0.05$ was used to select features.
