# Supplementary material for: Prognostic relevance of acquired uniparental disomy in serous ovarian cancer
Source: Mol Cancer. 2015 Feb 3;14(1):29. doi: 10.1186/s12943-015-0289-1 (PMC4320828; doi:10.1186/s12943-015-0289-1)
Supplement: Additional file 8: Table S4. — Association between frequency of aUPD and mutation status of TP53, BRCA1, BRCA2, homolog recombination (HR) and double-strand break (DSB) genes and aUPD regions at 17q (A-H), 22q (A-D), TP53, BRCA1 and BRCA2. [file 12943_2015_289_MOESM8_ESM.pdf]

Table S4. Association between frequency of aUPD and mutation status of *TP53*, *BRCA1*, *BRCA2*, homolog recombination (HR) and double-strand break (DSB) genes and aUPD regions at 17q (A-H), 22q (A-D), *TP53*, *BRCA1* and *BRCA2*.

Comparison	*p*	*q*
Somatic mutation at *TP53* vs no mutation*	0.004	**0.008**
Germline mutation at BRCA1 vs no mutation	0.052	0.069
Germline mutation at BRCA2 vs no mutation	0.101	0.127
Somatic mutation at BRCA1 vs no mutation	0.871	0.871
Somatic mutation at BRCA2 vs no mutation	0.290	0.331
Mutation at BRCA1 (somatic & germline) vs no mutation	0.107	0.129
Mutation at BRCA2 (somatic & germline) vs no mutation	0.046	0.069
Somatic mutation at HR and DSB genes vs no mutation	0.825	0.860
aUPD at *TP53* vs no aUPD	2.04×10^{-6}	$\mathbf{4.89 \times 10^{-5}}$
aUPD at *BRCA1* vs no aUPD	6.54×10^{-5}	**0.0003**
aUPD at *BRCA2* vs no aUPD	0.021	**0.0383**
aUPD at 17qA vs no aUPD	8.84×10^{-5}	**0.0004**
aUPD at 17qB vs no aUPD	1.76×10^{-5}	**0.0002**
aUPD at 17qC vs no aUPD	2.27×10^{-5}	**0.0002**
aUPD at 17qD vs no aUPD	0.0002	**0.0007**
aUPD at 17qE vs no aUPD	0.0005	**0.0012**
aUPD at 17qF vs no aUPD	0.0003	**0.0008**
aUPD at 17qG vs no aUPD	5.90×10^{-5}	**0.0003**
aUPD at 17qH vs no aUPD	0.0003	**0.0009**
aUPD at 22qA vs no aUPD	0.0494	0.0688
aUPD at 22qB vs no aUPD	0.0021	**0.0046**
aUPD at 22qC vs no aUPD	0.0430	0.0688
aUPD at 22qD vs no aUPD	0.0239	**0.0410**

*Kruskal-Wallis test was performed to test association between frequency of aUPD and *TP53* mutation. The remaining comparisons were performed by using Wilcox-rank sum test. *q*, Benjamini-Hochberg's FDR.$q < 0.05$ was used to select features.
